# Sample-level enrichment analysis unravels shared stress phenotypes among multiple cancer types

**DOI:** 10.1186/gm327

**Published:** 2012-03-29

**Authors:** Gunes Gundem, Nuria Lopez-Bigas

**Affiliations:** 1 Research Unit on Biomedical Informatics, Department of Experimental and Health Sciences, Universitat Pompeu Fabra, Dr. Aiguader 88, Barcelona, Spain; 2 Wellcome Trust Sanger Institute, Wellcome Trust Genome Campus, Hinxton, Cambridge, CB10 1SA, UK; 3 Institució Catalana de Recerca i Estudis Avançats (ICREA), Passeig Lluis Companys 23, 08010, Barcelona, Spain

## Abstract

**Background:**

Adaptation to stress signals in the tumor microenvironment is a crucial step towards carcinogenic phenotype. The adaptive alterations attained by cells to withstand different types of insults are collectively referred to as the stress phenotypes of cancers. In this manuscript we explore the interrelation of different stress phenotypes in multiple cancer types and ask if these phenotypes could be used to explain prognostic differences among tumor samples.

**Methods:**

We propose a new approach based on enrichment analysis at the level of samples (sample-level enrichment analysis - SLEA) in expression profiling datasets. Without using *a priori *phenotypic information about samples, SLEA calculates an enrichment score per sample per gene set using z-test. This score is used to determine the relative importance of the corresponding pathway or module in different patient groups.

**Results:**

Our analysis shows that tumors significantly upregulating genes related to chromosome instability strongly correlate with worse prognosis in breast cancer. Moreover, in multiple tumor types, these tumors upregulate a senescence-bypass transcriptional program and exhibit similar stress phenotypes.

**Conclusions:**

Using SLEA we are able to find relationships between stress phenotype pathways across multiple cancer types. Moreover we show that SLEA enables the identification of gene sets in correlation with clinical characteristics such as survival, as well as the identification of biological pathways/processes that underlie the pathology of different cancer subgroups.

## Background

Complex genetic diseases such as cancer are characterized by phenotypic heterogeneity reflected at the molecular level in the form of variations in the activity of certain signaling pathways. In support of this notion, recent cancer genome studies point to the idea that distinct types of alterations in different genes tend to accumulate in pathways central to the control of cell growth and cell fate determination [1-4]. It has been proposed that expression signatures indicative of activity status of pathways can be used to define specific molecular phenotypes that characterize individual tumors [5]. A number of methods have been developed to analyze the transcriptomic changes specific to tumor samples and identify patterns of pathway deregulation that differentiate distinct patient subgroups [6-12]. These methodologies are based on the idea that analysis of pathway-level differences among samples could have an advantage of reflecting the true oncogenic phenotypes achieved through consistent expression of a set of genes compared with the acute expression of a single gene. However, each of these methods has been designed to address specific questions and, thus, have limited use for a more general application. For instance, that of Xia and Wishart is specific to metabolomic data [9], and that of Bild *et al*. [6] requires cell line perturbation data in a platform comparable to that of the tumor data. The methodologies developed by Edelman *et al*. [7], Verhaak *et al*. [8] and Yi *et al*. [10] require *a priori *information of phenotypic classification of the samples. In this manuscript, we propose a new methodology, sample-level enrichment analysis (SLEA), that overcomes these limitations and has a more general use for enrichment analysis (EA) at the level of samples. The pathways or modules are represented as lists of genes, which can be obtained from literature or online repositories such as Gene Ontology, as well as determined through other high-throughput assays. Without using *a priori *phenotypic information about the samples, SLEA calculates an enrichment score per sample per gene set using z-test. This score is used to determine the relative importance of the corresponding module or pathway in different patient groups. We use this approach to test the hypothesis described in the following paragraph.

It has been proposed that, during the progression of cancer, the capacity of cancer cells to survive in the hypoxic and nutrient-deprived tumor microenvironment is a crucial step towards malignancy [13]. Adaptation to survival under these stress signals can override normal cellular stress responses, leading to the persistence and progression of the carcinogenic phenotype. Different types of stress insults, such as senescence-induced, metabolic, and oxidative, represent a common set of oncogenesis-associated cellular barriers that cancer cells must tolerate through stress support pathways [14]. For example, to overcome the senescence barrier, malignant cells have been proposed to deregulate proteins in senescence-mediating pathways such as Rb signaling. These alterations are collectively referred to as the stress phenotypes of cancers [14]. In this study, we asked if stress phenotypes of tumor samples could be used to explain their prognostic differences. To this end, we used publicly available gene expression profiles of patient cohorts of different types of cancers and gene signatures related to different stress phenotypes. We performed EA in each tumor sample in each patient cohort in order to detect differentially enriched modules. We show that EA with a chromosomal instability (CIN)-related gene signature has prognostic power in some cancer types but not in others. In all cancer types, however, patient sup-groups positively enriched for the same gene set shared key properties related to their stress phenotypes, indicating dependence of these tumors in certain stress support pathways.

## Materials and methods

### Transcriptomic data

We collected 11 publicly available expression profiling datasets from the Gene Expression Omnibus (GEO) and TCGA data portal [1,6,15-23] (Table 1). Each dataset consists of microarray expression data for primary tumors. We selected as datasets to include those that are on a single-channel platform, have survival information and contain more than 81 patients (see 'Robustness analysis' section below). The sample number varies from 111 to 766 across all datasets. Before EA, the data were pre-processed as follows (raw data were downloaded for all datasets). For Affymetrix data (9/11 datasets), CEL files were processed and normalized using the rma function in the 'affy' package [24] from R Bioconductor [25]. The result of normalization is log2-transformed absolute readings. For non-Affy experiments (2/11), expression data were normalized using the vsn normalization method from R Bioconductor [25]. After normalization, the input data were obtained by median-centering the expression value of each gene across all the samples (row median) and dividing the value by the standard deviation (row standard deviation). The expression value obtained in this step is a measure of how much a gene is expressed in a sample compared to all the other samples in the dataset. Hence, the heterogeneity and number of the tumor samples in the dataset affect the relative expression values. The stratification of the samples based on their enrichment patterns and the interpretation of this stratification, therefore, is sensitive to the clinical characteristics of the samples in the dataset. For example, the meaning of the median-centered expression value is different if the dataset includes normals in addition to cancer samples compared to if it includes tumor samples only. The selection of datasets should be done taking into account the type of question to be addressed. With this in mind, in our study, we include datasets that contain primary tumor samples only in order to answer the question of which modules/pathways are differentially enriched among different groups of samples of the same tumor type. All datasets used are provided on the SLEA website [26].

**Table 1:** Tumor profiling data sets used in the study

Name	Tumor type(s)	Sample number	Source
Ivshina *et al*. 2006 [21]	Breast	289	GEO: GSE4922
Pawitan *et al*. 2005 [19]	Breast	159	GEO: GSE1456
Wang *et al*. 2005 [15]	Breast	286	GEO: GSE2034
Kim et al. 2010 [20]	Bladder	257	GEO: GSE13507
TCGA 2008 [1]	Brain	400	TCGA: glioblastoma
Tothill *et al*. 2008 [16]	Ovary	284	GEO: GSE9891
Crijns *et al*. 2009 [22]	Ovary	416	GEO: GSE13876
TCGA, 2011 [23]	Ovary	521	TCGA: ovarian serous
Bild *et al*. 2006 [6]	Lung	112	GEO: GSE3141
Raponi *et al*. 2006 [18]	Lung	131	GEO: GSE4573
Smith *et al*. 2010 [17]	Colon	233	GEO: GSE17538

Each dataset contains a number of patients with survival information. The 'Source' column gives the GEO accession id of the experiment.

### Gene modules

Gene modules (gene sets) were collected from Gene Ontology [27], MSigDB [28] and the supplementary datasets of the indicated publications (Table 2). Using Gitools [29], we performed overlap analysis between the modules used. Some modules from Gene Ontology and MsigDB have high overlap (Jaccard index > 0.25) (Figure S1 in Additional file 1). We interpreted the results taking this into consideration. All modules used are provided on the SLEA website [26].

**Table 2:** List of modules extracted from expression data

Name	Description	Number of genes	Reference
CIN genes	A signature of genes upregulated in chromosomal instability and predictive of clinical outcome	70	[35]
Rb-E2F targets	Rb-E2F interaction network built computationally using protein interaction databases	147	[43]
Down in senescence bypass	Genes downregulated in fibroblasts that bypass RAS-induced senescence	3, 030	[37]
Up in senescence bypass	Genes upregulated in fibroblasts that bypass RAS-induced senescence	2, 714	[37]
Down in senescence	Genes downregulated in fibroblasts in replicative senescence	6, 122	[42]
Up in senescence	Genes upregulated in fibroblasts in replicative senescence	6, 048	[42]
Pujana ATM network	Computational network around Atm built using expression profiling and functional and genomic data	1, 041	[45]
Pujana BRCA1 network	Computational network around Brca1 built using expression profiling and functional and genomic data	1, 198	[45]
Pujana BRCA2 network	Computational network around Brca2 built using expression profiling and functional and genomic data	305	[45]
Pujana CHEK2 network	Computational network around Chek2 built using expression profiling and functional and genomic data	559	[45]
Pujana XPRSS network	Computational network around Xprss built using expression profiling and functional and genomic data	118	[45]
Bortezomib treatment DOWN	Genes downregulated in cancer cells treated with bortezomib	1, 769	[47]
Bortezomib treatment UP	Genes upregulated in cancer cells treated with bortezomib	1, 278	[47]
Eeyarestatin treatment DOWN	Genes downregulated in cancer cells treated with eeyarestatin	2, 170	[47]
Eeyarestatin treatment UP	Genes upregulated in cancer cells treated with eeyarestatin	2, 062	[47]
Downreg in PI3K-hyper	Genes downregulated in Rb-deficient breast cancer cell line treated with rapamycin	100	[49]
Upreg in Pi3K-hyper	Gene upregulated in hormone therapy-resistant breast cancer	1, 475	[49]
PTEN mutation signature	PTEN mutation signature upregulated in PTEN-mutant breast cancer	592	[50]
Up in TSC1 mTORC1	Genes upregulated in Tsc1-/- mutant versus WT MEFs	167	[51]
Down in TSC1 mTORC1	Genes downregulated in Tsc1-/- mutant versus WT MEFs	101	[51]

All the modules extracted from gene expression profiling data and used in the study. MEF, mouse embryonic fibroblast; WT, wild type.

### Sample-level enrichment analysis

**Figure 1:**
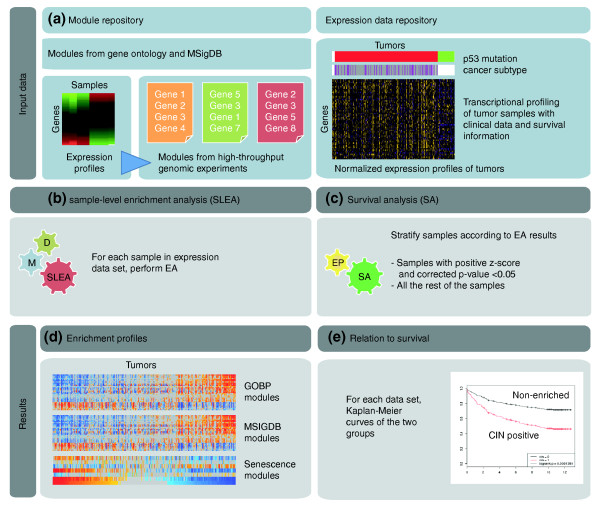
**General schema of the approach**. First, module and expression data repositories are created. **(a) **Left: gene modules were obtained from a number of sources such as Gene Ontology and MSigDB as well as from expression datasets listed in Table 2. Right: high-throughput expression profiling experiments of tumor samples with clinical information were normalized by median-centering the expression value of each gene across all the samples and dividing the value by the standard deviation. In the heat map, purple color indicates low expression while yellow means high expression. **(b) **The first step in the pipeline is sample-level enrichment analysis (SLEA) of the dataset with each of the modules (M) in each of the datasets (D). **(c) **The second step is survival analysis according to the enrichment pattern (EP) for a module. **(d) **The results of the enrichment analysis (EA) can be visualized in Gitools as heat maps. Red indicates significant upregulation of the module while blue indicates significant downregulation. Grey is for non-significant results. GOBP, Gene Ontology biological process. **(e) **Differentially enriched modules are studied for their correlation to some clinical feature, in this case, survival. Shown here are Kaplan-Meier curves of samples with two different enrichment patterns.

EA for each sample in each dataset was performed using Gitools [29,30] (Figure 1b). Gitools is a java application for genomic data analysis and visualization the main distinctive feature of which is that data and results are represented using interactive heat maps. Among other tests, Gitools provides different statistical methods to assess the enrichment of gene modules in high-throughput genome-wide profiling data. The main advantage of Gitools for the type of analysis presented in this manuscript is that it can perform many EAs (one per sample and module in this case) in one single run and the results are provided in the form of interactive heat maps, which are useful to compare the results between different samples and different modules. Modules can be literature-based as well as consist of sets of genes obtained through analysis of other types of genome-wide studies. In this study, we used the z-score method as described previously [31]. This method compares the mean (or median) expression value of genes in each module to a distribution of mean (or median) of 10, 000 random modules of the same size drawn from the expression values for the same sample. The result of this EA is a z-score, which is a measure of the difference between the observed and expected mean (or median) expression values for a gene set. The *P*-value related to each z-score is automatically corrected for multiple testing using the Benjamini-Hochberg method [32]. We define modules as 'positively enriched' in a sample if they have a positive z-score and a corrected *P*-value < 0.05, and 'non-enriched' otherwise. The results are visualized as heat maps of z-scores in Gitools, which is useful for the identification and interpretation of enrichment patterns among samples.

### Survival analysis

We used the 'coxph' function from the 'survival' package of R [25] (Figure 1c). In survival analysis with the CIN signature, the survival data of the samples with positive enrichment for the signature (positive z-scores with corrected *P*-value < 0.05) are compared to all the rest of the samples (non-enriched) in the dataset. For the survival analysis related to upregulation of the two-gene signature (CDKN2A and MKI67) [33], we compare the samples with an expression value greater than the standard deviation of the row for both genes to all the rest of the samples in the dataset.

### Web server

To facilitate the representation and interpretation of the results generated by our analyses, we created a web service using Onexus [34] that allows navigation of all the heat maps and details of the statistical results for each of the dataset and modules analyzed along with the datasets included in the analysis [26].

### Technical consideration of SLEA and robustness analysis

Some considerations of the SLEA approach as presented here are important to take into account. First, the z-test requires normality on data. Since SLEA uses the distribution of means of random sets of genes, due to the central limit theorem, even if the expression data do not follow normal distribution, the distribution of the sample mean is normal provided that the number of permutations is large (we use 10, 000 permutations). The distribution of the sample median, on the other hand, may not be normal, although for large numbers of permutations it is usually close to it. However, the median is a measure more robust to outliers; hence, we performed the same EAs with sample mean and median separately and compared the results. The z-scores obtained with the different test statistics are almost identical (r = 0.99) (Figure S2 in Additional file 1). We use the median for all the plots and results of EA shown in the manuscript.

The second important consideration is the robustness of SLEA with regard to changes in the cohort and how it is affected by the sizes of the datasets (that is, the number of samples included). To assess how this influences the results obtained with SLEA and to identify the number of samples under which our methodology works best, we devised a random sampling procedure (Figure S3 in Additional file 1). Using three datasets (Table 1), GSE4922 ([GEO:GSE4922]; breast cancer dataset with 289 tumor samples), TCGA-OV (ovarian cancer dataset with 521 samples) and GSE4573 ([GEO:GSE4573]; lung cancer dataset with 131 samples), we generated different populations of random datasets with the same number of samples. The sample size ranged from 11 to 201 with an increment of 10 for GSE4922 [GEO:GSE4922] and TCGA-OV datasets. For the smallest dataset, it was from 11 to 111 with an increment of 10. Each population contained 100 datasets producing a total of 2, 000 datasets for GSE4922 [GEO:GSE4922] and TCGA-OV and 1, 100 datasets for GSE4573 [GEO:GSE4573] (Figure S3 in Additional file 1). For each of those random datasets we performed median centering followed by the median z-test EA for the CIN signature. Next we performed correlations of the obtained z-scores for each pair of random datasets in each population and plotted box-and-whisker plots of correlation coefficients for each of the dataset sizes (Figure S3 in Additional file 1). This analysis shows that, for datasets with more than 71 samples, the correlations are always higher than 0.99 (Figure S4 in Additional file 1). We also did a *t*-test comparing the z-scores of all the samples in a population to the z-scores the same sample has in the population with the greatest number of samples (201 samples for GSE4922 [GEO:GSE4922] and TCGA-OV and 111 for GSE4573 [GEO:GSE4573]). This analysis shows that the proportion of samples that are significantly different (*t*-test corrected *P*-value < 0.05) is less than 0.05 for sample sizes greater than 81. In summary, we can conclude that SLEA results are highly robust for datasets with 81 or more samples.

## Results and discussion

In this study, we aim to demonstrate the use of the SLEA approach by detecting the biological processes underlying the differences between clinically distinct patient subgroups. To do this, we performed SLEA using Gitools [29] for 11 cancer datasets with various relevant gene sets (Tables 1 and 2). Gitools provides two main advantages for this type of analysis, i) one single run of Gitools is enough to perform EA for a large number of samples and modules, and ii) the results are shown in the form of an interactive heat map, which facilitates the comparison between samples and gene sets, and the interpretation of the results. For the sake of clarity and space considerations, we focus on the results for one breast cancer dataset (GSE4922 [GEO:GSE4922]; Table 1) and we point to similarities with and differences from the rest of the datasets, for both breast and other cancer types. The results of the 11 datasets along with the statistical details are accessible at the web service [26] and some results are shown as supplementary figures in Additional file 1.

### Stratification of patient cohorts in breast cancer

**Figure 2:**
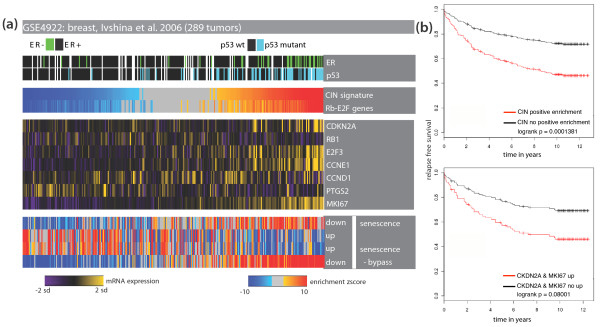
**Enrichment with the CIN signature reflects the response status to oncogene-induced stress and has prognostic power in breast cancer**. **(a) **Heat map of tumor samples as columns and genes or modules as rows. Enrichment with gene modules is shown with colors from blue (downregulation) to red (upregulation) while gray values indicate no significant deviation from the expected median value. Samples are ordered according to the z-score of the chromosomal instability (CIN) signature. (Note that a sample might be insignificant (shown with gray) even if it has a z-score greater than the adjacent non-gray sample. This is because multiple testing correction is done for each sample independently.) Expression levels of genes are shown in colors from purple (low expression) to yellow (high expression). The samples with upregulation of the CIN signature include those that upregulate *CDKN2A *with concomitant down-regulation of *RB1*. Deregulation of Rb signaling in these samples is reflected in their over-expression of the Rb-E2F target genes *CCNE1 *and *E2F3*. These samples show a transcriptional program opposite to what is observed in senescent cells, indicating that they activated transcriptional programs indicative of senescence bypass. Shown is a breast cancer dataset by Ivshina *et al*. [21] (289 samples). **(b) **Kaplan-Meier curves for breast cancer patients. The red curve is for the samples with high expression of *CKDN2A *and *MKI67 *(lower panel) and positive enrichment of the CIN gene signature (upper panel). The black curves are for the rest of the samples in the dataset. It was shown that the concomitant expression of *CDKN2A *and *MKI67 *is related to impairment of the Rb pathway, hence to subsequent tumor events in ductal carcinoma *in situ *of the breast. The CIN gene signature has stronger prognostic power than the two-gene signature.

Focusing on the three breast cancer datasets, we first aimed to stratify the tumors in each cohort by performing EAs with a CIN-related gene signature previously shown to predict clinical outcome in multiple tumor types [35]. In all the datasets, based on the EA results, we separated the tumors into two groups: positively enriched (positive z-scores with corrected *P*-value < 0.05) and non-enriched (all the rest of the samples that did not satisfy the criteria) (Figure 2; Figure S5 in Additional file 1; online supporting material [26]). Subsequent survival analysis showed that the first group had worse survival than the second group in all the breast cancer datasets analyzed (Figure 2b; Figure S5 in Additional file 1). Moreover, the tumors in the first group coincided with more aggressive subtypes of breast cancer (luminal B and basal-like) [36] (Figure S5 in Additional file 1) and p53 mutation carriers [36] (Figure 2a). These results show that our EA approach can be used to stratify patients with respect to a clinical property, in this case survival. We refer to the tumors with significant upregulation of the CIN signature as 'CIN-positive' in the rest of the manuscript.

### CIN-positive tumors activate a senescence-bypass transcriptional program

Senescence is an important tumor suppressive barrier to the progression of cancer [37-41]. Molecular markers of senescence are observed in pre-malignant lesions while they are lost in the malignant counterparts [37-41]. Prompted by this idea, we set out to compare the CIN-positive tumors to the non-enriched tumors in terms of their expression of senescence-related transcriptional programs. We performed EA with genes that are differentially regulated in fibroblasts undergoing replicative senescence (with the modules named 'down and up in senescence') [37] and in fibroblasts that bypass RAS-induced senescence (with the modules named 'down and up in senescence-bypass') [42]). Indeed, in all breast cancer datasets, the primary tumors with the CIN signature were enriched for the senescence-bypass-related transcriptional program while they exhibited expression patterns opposite to that observed during senescence (Figure 2; online supporting material [26]). Furthermore, we checked the expression level of the genes *CDKN2A *and *MKI67*, biomarkers indicative of an abrogated response to senescence-inducing stimulus [33]. These markers were previously shown to indicate compromised Rb signaling and predict subsequent tumor events in breast cancer patients diagnosed with ductal carcinoma *in situ *[33]. Indeed, some of the CIN-positive tumors displayed concomitant over-expression of CDKN2A and MKI67 together with Rb targets CCNE1 and E2F3 (Figure 2; online supporting material [26]), indicating deregulation of the Rb pathway. As a better measure of Rb signaling status, we used a set of genes repressed by Rb-E2F (with the module name 'Rb-E2F genes') when Rb signaling is functional [43]. EA with this gene signature confirmed that, although the overlap between the two signatures is low (Jaccard index = 0.22), CIN-positive breast tumors have positive enrichment for Rb-E2F targets, and thus have signs of compromised Rb signaling (Figure 2; online supporting material [26]). All these results indicate that CIN-positive tumors have activated transcriptional programs indicative of an abrogated response to senescence.

Finally, we compared the prognostic power of the CIN signature to that of concomitant overexpression of *CDKN2A *and *MKI67 *(positive normalized expression values for both genes in the same sample) [33]. As seen in Figure 2 (Figure S5 in Additional file 1; online supporting material [26]), the CIN signature is more informative than the two-gene signature (smaller *P*-values). As many samples with upregulation of the CIN signature have p53 mutations, we sought to determine if the prognostic power of the CIN signature is independent of p53 mutation status. We performed survival analysis in the datasets with p53 mutation status information excluding the tumors with p53 mutations. Of 289 tumors, 189 had wild-type p53 in the GSE4922 dataset [GEO:GSE4922]. In breast cancer, enrichment with the CIN signature is strongly related to bad prognosis even among samples with wild-type p53, indicating that indeed the predictive power of this signature is independent of p53 mutation (Figure S6 in Additional file 1).

### Stress phenotypes of the CIN-positive tumors

**Figure 3:**
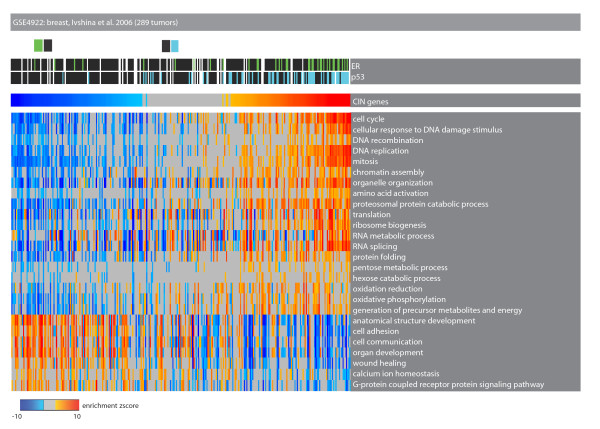
**Enrichment analysis of Gene Ontology biological process terms in tumors with upregulation of the CIN signature**. Heat map of tumor samples as columns and Gene Ontology biological process terms as rows. Color codes are as described in Figure 2. The top part of the heat map shows the p53 mutation and estrogen receptor (ER) status in each sample. The samples with upregulation of the CIN signature significantly upregulate genes annotated with 'cell cycle', 'DNA replication' and downregulate genes related to 'cell adhesion', 'cell communication', and so on.

Next we performed EA with all Gene Ontology biological process terms in order to identify the biological properties characterizing CIN-positive tumors. These tumors significantly downregulate genes related to processes such as 'cell communication' and 'wound healing' (Figure 3; online supporting material [26]). This is in agreement with previous observations showing that the upregulation of a wound response signature is inversely correlated with good prognosis [44].

**Figure 4:**
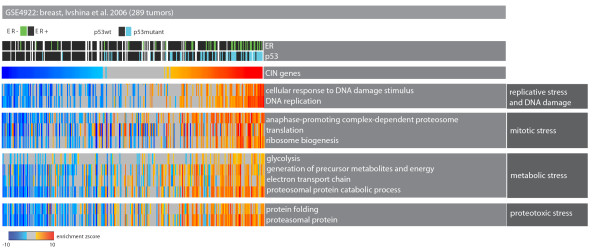
**Tumors with upregulation of the CIN signature have distinctive stress phenotypes**. Heat map of tumor samples as columns and Gene Ontology biological process terms as rows. Color codes are as described in Figure 2. The top part of the heat map shows the p53 mutation and estrogen receptor (ER) status in each sample. The samples with up-regulation of the CIN signature significantly upregulate genes related to important stress support mechanisms such as 'mitotic stress', 'proteotoxic stress', and so on.

On the other hand, some categories such as 'cellular response to DNA damage', 'protein folding' and 'translation' were significantly upregulated. We argue that this transcriptional program can be explained by non-oncogene addiction, which is defined as the dependence of cancer cells on stress support pathways that are not themselves tumorigenic [14]. Most of the differentially enriched Gene Ontology terms can be attributed to one of these stress support pathways: 'DNA damage and replicative stress', 'mitotic stress', 'proteotoxic stress' and 'metabolic stress' (Figure 4; online supporting material [26]). The deregulation of these pathways might be indicative of non-oncogenic vulnerabilities of the CIN-positive tumors.

### Dependence on DNA damage signaling

**Figure 5:**
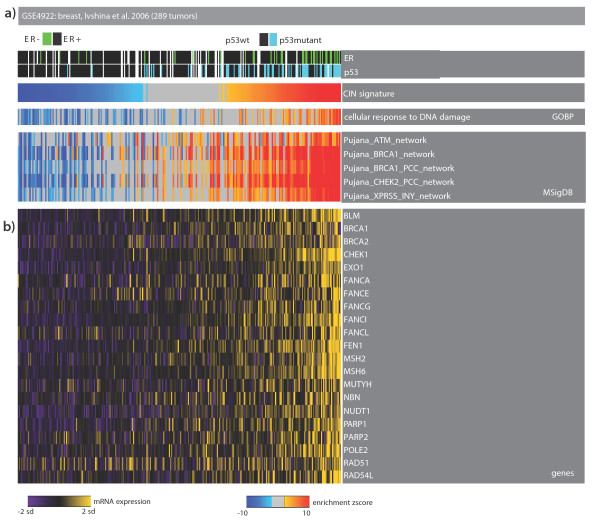
**Tumors with upregulation of the CIN signature are addicted to DNA repair**. **(a) **Heat-map of tumor samples as columns and genes or modules as rows. Color codes are as described in Figure 2. The top part of the heat map shows the p53 mutation and estrogen receptor (ER) status in each sample. The samples with up-regulation of the CIN signature significantly upregulate genes annotated with DNA damage/repair-related categories and gene signatures related to DNA damage signaling network around ATM-BRCA1 (from MSigDB). **(b) **Selected genes involved in DNA damage repair.

We performed EA with selected gene modules from MSigDB. CIN-positive tumors, which are positively enriched for sets of genes related to mitotic checkpoint, anaphase- promoting complex, DNA damage response, are also enriched for networks of genes built computationally around key repair proteins (MSigDB modules from Pujana *et al*. [45]) (Figure 5; and online supporting material). Moreover, compared to other tumor samples, these tumors have higher expression levels of DNA repair/DNA damage response genes, including *PARP1/2 *and *BRCA1/2 *(Figure 5; online supporting material). Higher expression of these genes indicates that these tumors are dependent on the DNA damage response as explained by non-oncogene addiction. This observation also points to ideas for specialized therapeutic strategies for these aggressive tumors, which are mainly basal-like and luminal B, based on the possible addiction of these tumors to DNA repair pathways. Indeed, very recently, it was shown that combination therapy of iniparib (a poly (ADP-ribose) polymerase (PARP) inhibitor) and chemotherapy, without significant increased toxic effects, improved the clinical benefit and survival of patients with metastatic triple-negative breast cancer, a majority of which are also basal-like [46].

### Dependence on proteotoxic stress mechanisms

**Figure 6:**
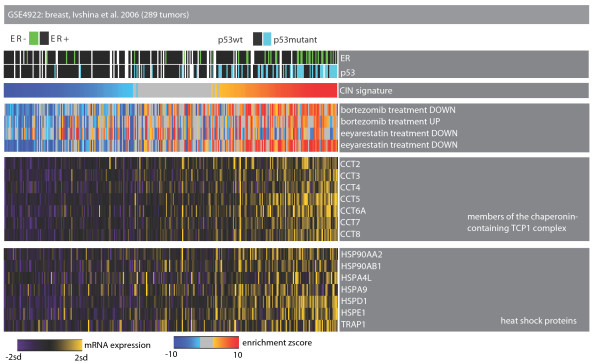
**Dependence on proteotoxic stress mechanisms**. Heat map of tumor samples as columns and genes or modules as rows. Color codes are as described in Figure 2. The top part of the heat map shows the p53 mutation and estrogen receptor (ER) status in each sample. The samples with up-regulation of the CIN signature significantly upregulate genes deregulated in cells treated with bortezomib and eeyarestatin.

We assessed the prevalence of proteotoxic stress mechanisms by performing an EA with sets of genes deregulated in cancer cell lines treated with bortezomib and eeyarestatin [47]. CIN-positive tumors significantly upregulated genes that increase in expression in response to both bortezomib, a proteasome inhibitor, and eeyarestatin, an inhibitor of endoplasmic reticulum-associated protein degradation (Figure 6; online supporting material [26]). At the gene level, these samples upregulated genes that are members of the chaperonin-containing complex and heat shock proteins. Of these genes, *HSP90 *complex is already a molecular target in cancer [48].

### Dependence on phosphoinositide 3-kinase/Akt signaling

**Figure 7:**
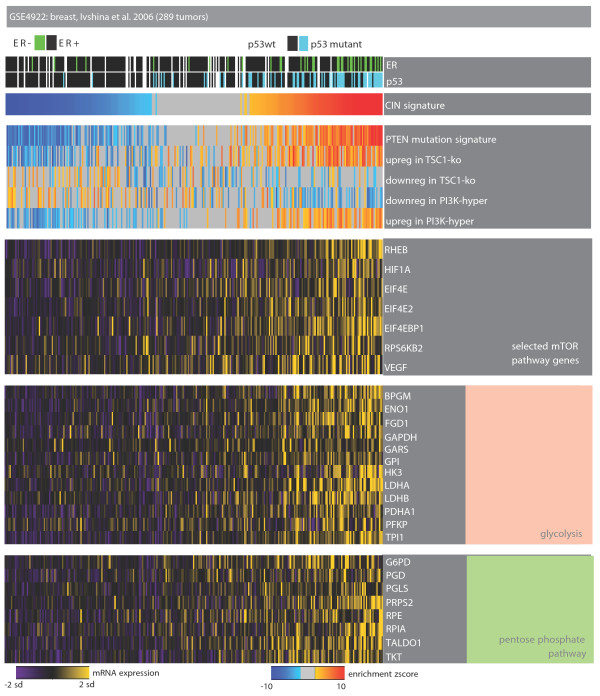
**Dependence on PI3K/Akt signaling**. Heat-map of tumor samples as columns and genes or modules as rows. Color codes are as described in Figure 2. The samples with positive enrichment for the CIN signature significantly upregulate gene signatures of activated PI3K-mTOR signaling. mTOR is an important modulator of metabolism and can be inhibited by rapamycin. The targets of mTOR are also upregulated in the same tumors. Hence, this dependence on mTOR could be exploited therapeutically.

CIN-positive tumors were also positively enriched for metabolism-related categories such as 'nucleotide metabolism', 'generation of precursor metabolites and energy', 'electron transport chain', 'ribosome biogenesis', and so on. Hence, we focused on a specific pathway that plays a crucial role in the regulation of cellular metabolism and its coupling to proliferation. We collected gene sets related to the phosphoinositide 3-kinase (PI3K)/Akt pathway and its downstream mammalian target of rapamycin (mTOR) signaling: 'genes deregulated in PI3K-hyper-activated, hormone resistant cells' [49] (modules named 'upreg and downred in PI3K-hyper'), 'PTEN mutation signature' [50] and genes deregulated in *TSC1 *knockout cells ('upreg in downreg in TSC1-ko') [51]. Figure 7 shows that the transcriptional program of tumors with the CIN signature is enriched for hyper-activated PI3K signaling as well as for genes upregulated in PTEN mutant cells. mTOR signaling activates the expression of genes encoding nearly every step of glycolysis and the pentose phosphate pathway, as well as critical enzymes in the *de novo *synthesis of sterols, isoprenoids, and fatty acids [51]. We used modules of genes regulated by mTORC1, a molecular complex that contains mTOR [51], to check if indeed the CIN-positive tumors also have activation of processes downstream of mTOR. As expected, the genes upregulated by mTORC1 are also upregulated in these samples (Figure 7; online supporting material [26]). mTORC1 promotes the expression of *HIF1A *[51]. In agreement with this, CIN-positive tumors overexpress *HIF1A *along with its target vascular endothelial growth factor (Figure 7; online supporting material [26]). As mTORC1 has been shown to induce the transcription of genes involved in important metabolic pathways [51], we checked the mRNA levels of enzymes from the glycolysis and pentose phosphate pathway. Indeed, most of these enzymes are upregulated in CIN-positive tumor samples (Figure 7; online supporting material [26]). Together these observations indicate that the CIN-positive tumors have activated signaling through mTOR. These results suggest two things. First, these tumors might be addicted to pathways related to metabolic stress in addition to DNA damage stress. If this is indeed the case, then, secondly, inhibitors of mTOR, such as rapamycin, might be useful for the treatment of these cancers. The observations in this and the previous section show that sample-level EA can help pinpoint pathway dependencies in different subgroups of tumors, which can be used to design rational therapeutic approaches specific to each group of patients.

### CIN-positive tumors indicate worse prognosis in breast cancer but not in other cancer types

**Figure 8:**
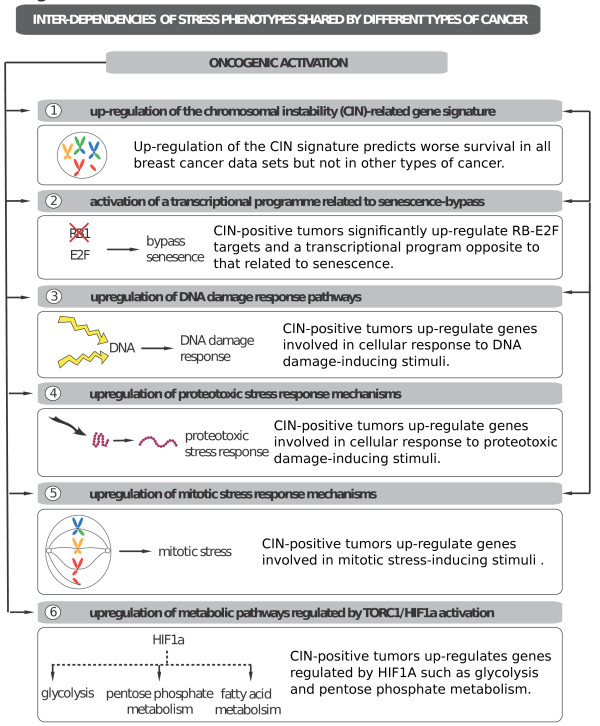
**Pathway interdependencies**. Oncogenic signaling causes stress through many different, interrelated mechanisms. These mechanisms include: 1) up-regulation of the CIN-related genes; 2) activation of transcriptional program related to senescence-bypass; 3) upregulation of DNA damage response pathways; 4) upregulation of proteotoxic stress response mechanisms; 5) upregulation of mitotic stress response mechanisms; and 6) upregulation of metabolic pathways regulated by TORC1/HIF1 activation.

In order to determine if we can see similar patterns in other types of cancers, we performed the same EAs in tumor datasets comprising different types of cancer (in total 11 datasets): brain, lung, ovary, bladder and colon. In all the datasets the enrichment of the CIN signature divided the samples into two (see online supporting material [26]). There were two datasets showing marginal predictive power for the CIN signature (GSE13507 [GEO:GSE13507] for bladder and GSE13876 [GEO:GSE13876] for ovarian cancer). The rest of the datasets did not show significant difference in survival between the tumors defined by upregulation of the CIN signature and the rest of the samples (Figure S7 in Additional file 1). Nonetheless, in all the datasets, the tumors with significant upregulation of the CIN signature also upregulated the senescence-bypass transcriptional program and exhibited similar stress phenotypes as observed in breast cancer datasets (Figure S8 in Additional file 1; online supporting material [26]), indicating that the pathway interdependencies observed in breast tumors are shared across different types of cancer (Figure 8).

## Conclusions

EA is an effective way to analyze the statistically significant gene sets obtained using high-throughput functional genomics data. In this work, we propose an alternative approach for the analysis of tumor genomics data to detect clinically relevant patient subgroups. Instead of finding genes differentially expressed between two groups, we identify differentially enriched modules by performing sample-level EA (SLEA). Our method does not require information related to phenotypic classification of samples and can directly take gene sets as input. Moreover, by comparing enrichment results with available clinical information, SLEA enables the understanding of pathways/processes that underlie the clinical phenotypes such as survival. We applied our methodology to test the prognostic power of a gene signature related to chromosomal instability and to study the prevalence of stress phenotypes in different patient subgroups defined by the expression of this gene signature. The tumors significantly upregulating this signature were strongly correlated with worse prognosis in the three breast cancer datasets studied, but not in other tumor types. In all cancer types, however, the tumors with positive enrichment for this gene signature displayed a transcriptional program pointing to evasion of the senescence barrier and particular stress phenotypes, indicating strong interdependencies between these different pathways and therapeutic vulnerabilities for the tumor.

## Abbreviations

CIN: chromosomal instability; GEO: Gene Expression Omnibus; mTOR: mammalian target of rapamycin; PI3K: Phosphoinositide 3-kinase; SLEA: sample-level enrichment analysis.

## Competing interests

The authors declare that they have no competing interests.

## Authors' contributions

GG and NL designed the study, interpreted the data and drafted the manuscript. GG performed all statistical and other data analysis. All authors have read and approved the manuscript for publication.

## Supplementary Material

Additional file 1 **Supplementary figures**. Supplementary figure 1: result of overlap analysis of the modules used. Heat map of the Jaccard indices for overlap analysis among modules used in this study. Pink cells indicate high overlap (Jaccard index ≥ 0.6) while light blue shows no overlap. Supplementary figure 2: comparison of z-score mean and z-score median. Scatter plot of z-scores obtained using mean and median as the test statistic in EA of the Ivshina *et al*. [21] dataset with the chromosomal instability (CIN) signature. Since the correlation is high and the median is more robust to outliers, it is used for the test statistic. Supplementary figure 3: robustness analysis of SLEA. Step 1: randomization procedure to test for the size of the dataset. Populations of random datasets were created from the three datasets GSE4922 ([GEO:GSE4922]; Ivshina *et al*. [21]), TCGA-OV (TCGA Nature 2011 [23]) and GSE4573 ([GEO:GSE4573]; Raponi *et al*. [18]). Each population contained 100 datasets of a fixed number of samples. For GSE4922 [GEO:GSE4922] and TCGA-OV, the sample number varied from 21 to 201, and for GSE4573 [GEO:GSE4573], from 11 to 111. Step 2: for each random dataset in each population, we performed EA with the CIN signature. Step 3: within each population, we performed pair-wise correlation analysis between all random datasets. Step 4: we plotted the distribution of Pearson's correlation values for all populations in a box-and-whisker plot. Correlation values get closer to 1 as sample size increases and are greater than 0.99 for populations of 71 or more. Supplementary figure 4: results of the robustness analyses. Robustness analysis for changes in the cohort was performed for three datasets. Shown here are the plots for them. For GSE4922 [GEO:GSE4922] and TGCA-OV, correlation coefficients for all datasets get closer to 1 as sample size increases. Among all three datasets, correlation is greater than 0.99 for datasets of size 71. Supplementary figure 5: predictive power of the CIN signature in other breast cancer datasets. In the other breast cancer datasets, EA with the CIN signature segregates the patients into two groups that difference according to survival. Clinical information available for each dataset is shown along with the results of EA with the CIN gene signature. The color code for EA results is the same as in Figure 2. The red curve is for the samples with positive enrichment of the CIN signature (cin = 1). These samples have worse survival compared to all the other samples in each dataset (cin = 0; black curve). Supplementary figure 6: the predictive power of the CIN signature is independent from p53 mutation status. Kaplan-Meier curves for a patient cohort of breast cancer ((GSE4922) [GEO:GSE4922]) with wild-type p53. The red curve is for the samples with positive enrichment of the CIN gene signature (cin = 1). These samples have worse survival compared to all the other samples in each dataset (cin = 0; black curve). Supplementary figure 7: predictive power of the CIN signature in other types of cancers. Kaplan-Meier curves for brain, ovarian, lung and bladder cancer patients. The red curve is for the samples with high positive enrichment of the CIN gene (cin = 1). The black curves are for the rest of the samples (cin = 0). The CIN gene signature has a prognostic power in none of the datasets. Supplementary figure 8: CIN-positive tumors have similar stress properties in different cancer types. The panels are for brain, ovarian, lung and bladder cancers. For each cancer type, six properties are shown. The color code for enrichment analysis results (red to blue) is the same as in Figure 2. The properties are 1) upregulation of chromosomal instability genes, 2) senescence-bypass program, 3) DNA and replicative stress response genes, 4) metabolic stress response genes, 5) mitotic stress response genes, and 6) proteotoxic stress response genes. Click here for file
