# Supplementary material for: Sample-level enrichment analysis unravels shared stress phenotypes among multiple cancer types
Source: Genome Med. 2012 Mar 29;4(3):28. doi: 10.1186/gm327 (PMC3446278; doi:10.1186/gm327)

Supplementary figure 1

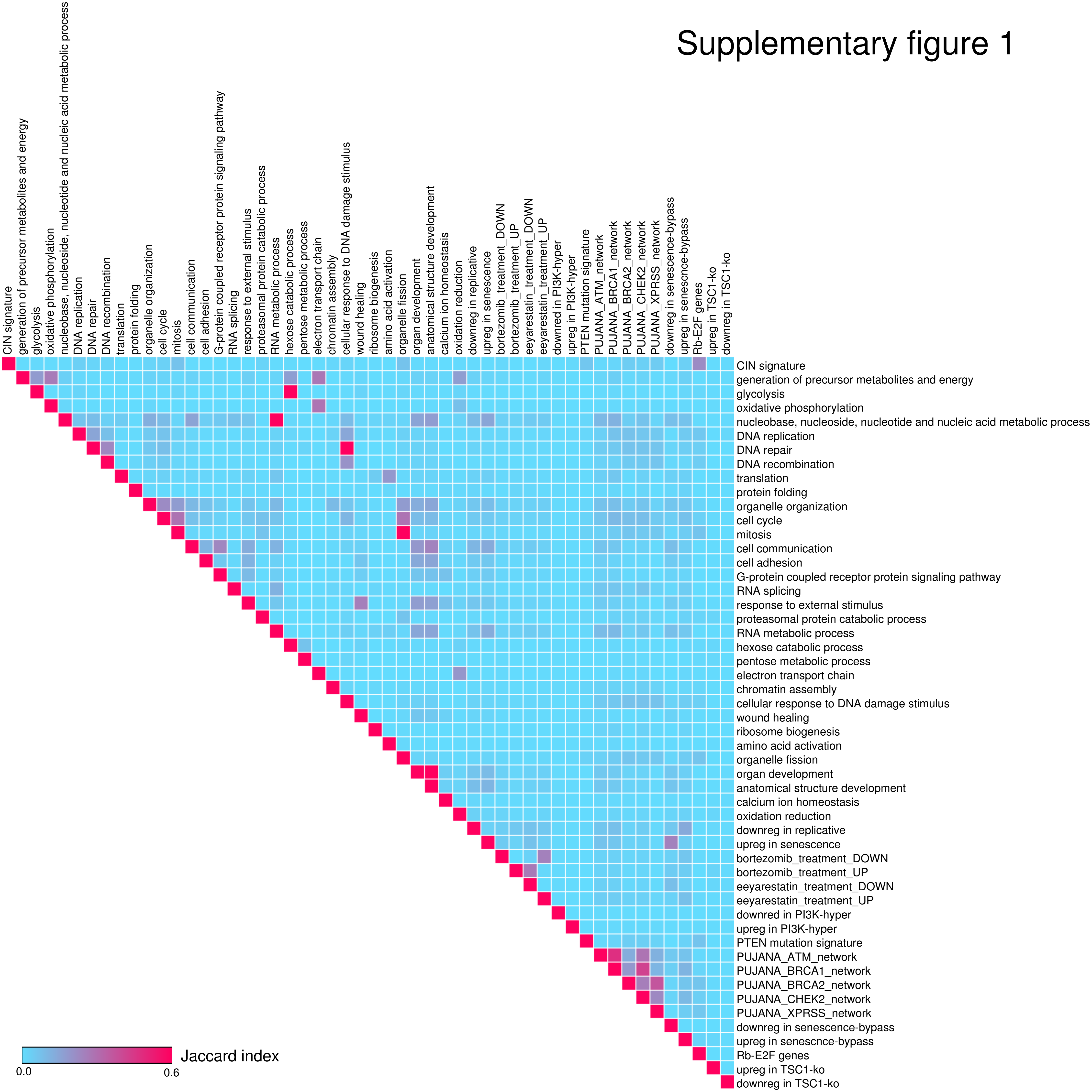

Supplementary figure 2

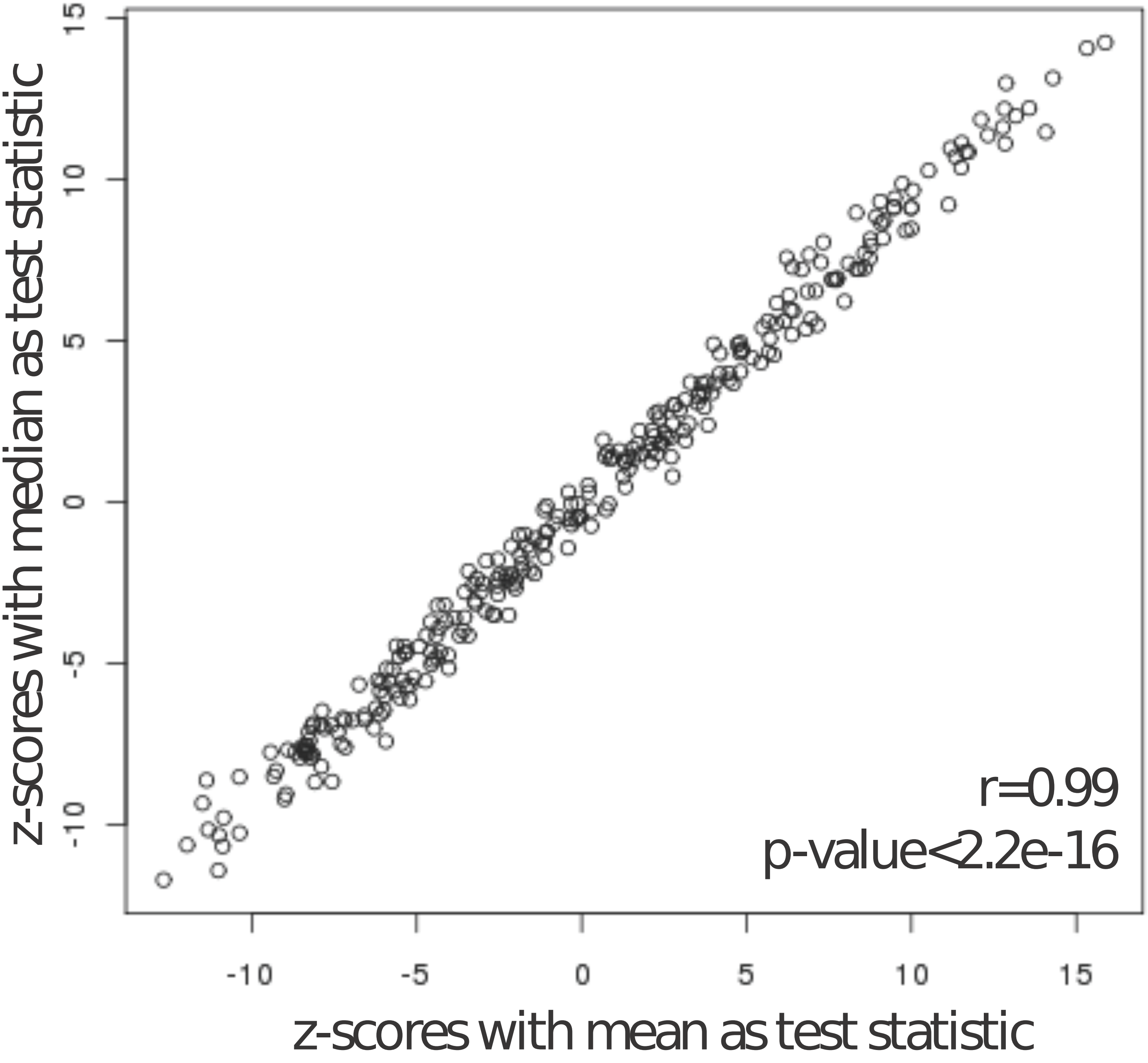

Supplementary figure 3

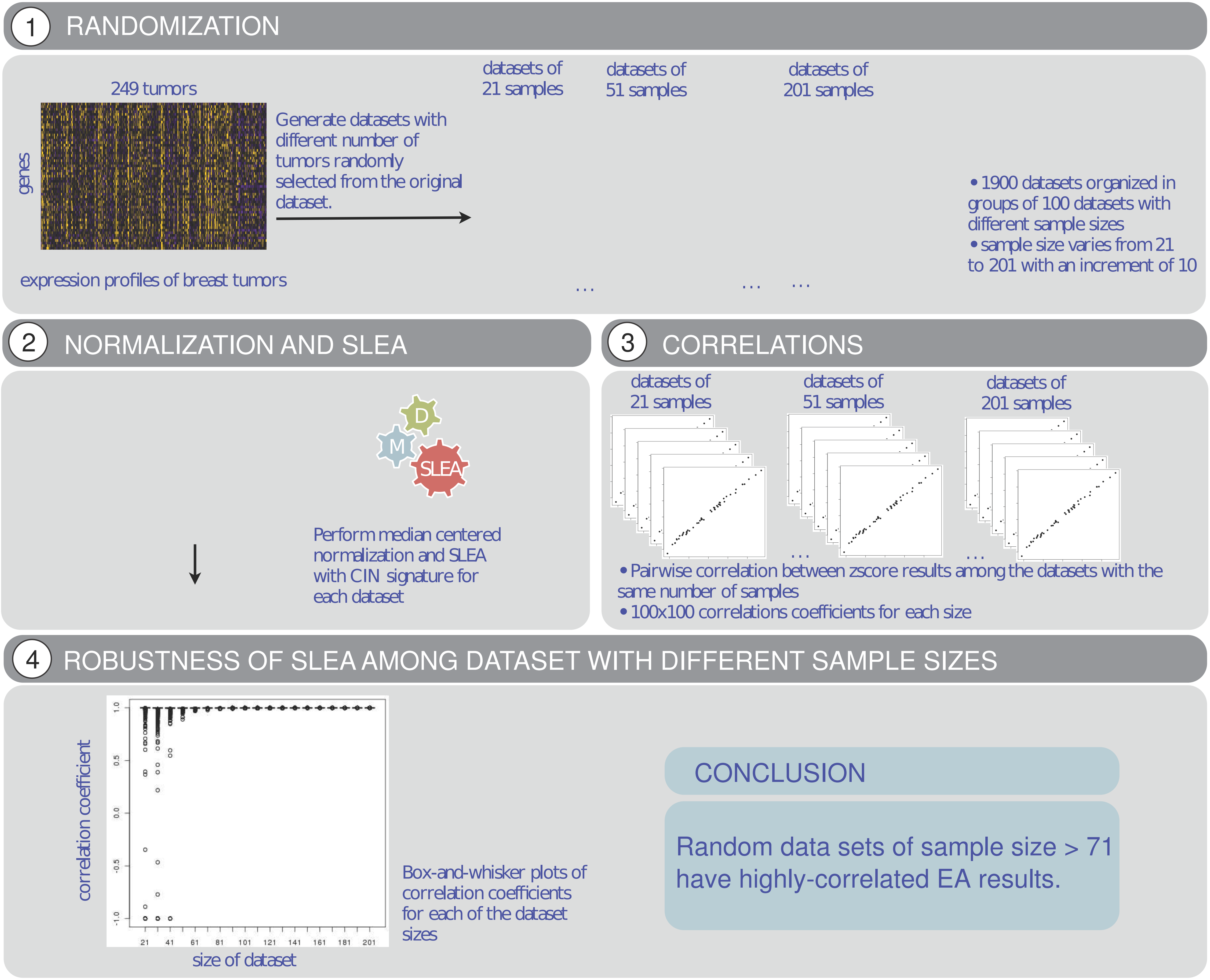

Supplementary figure 4

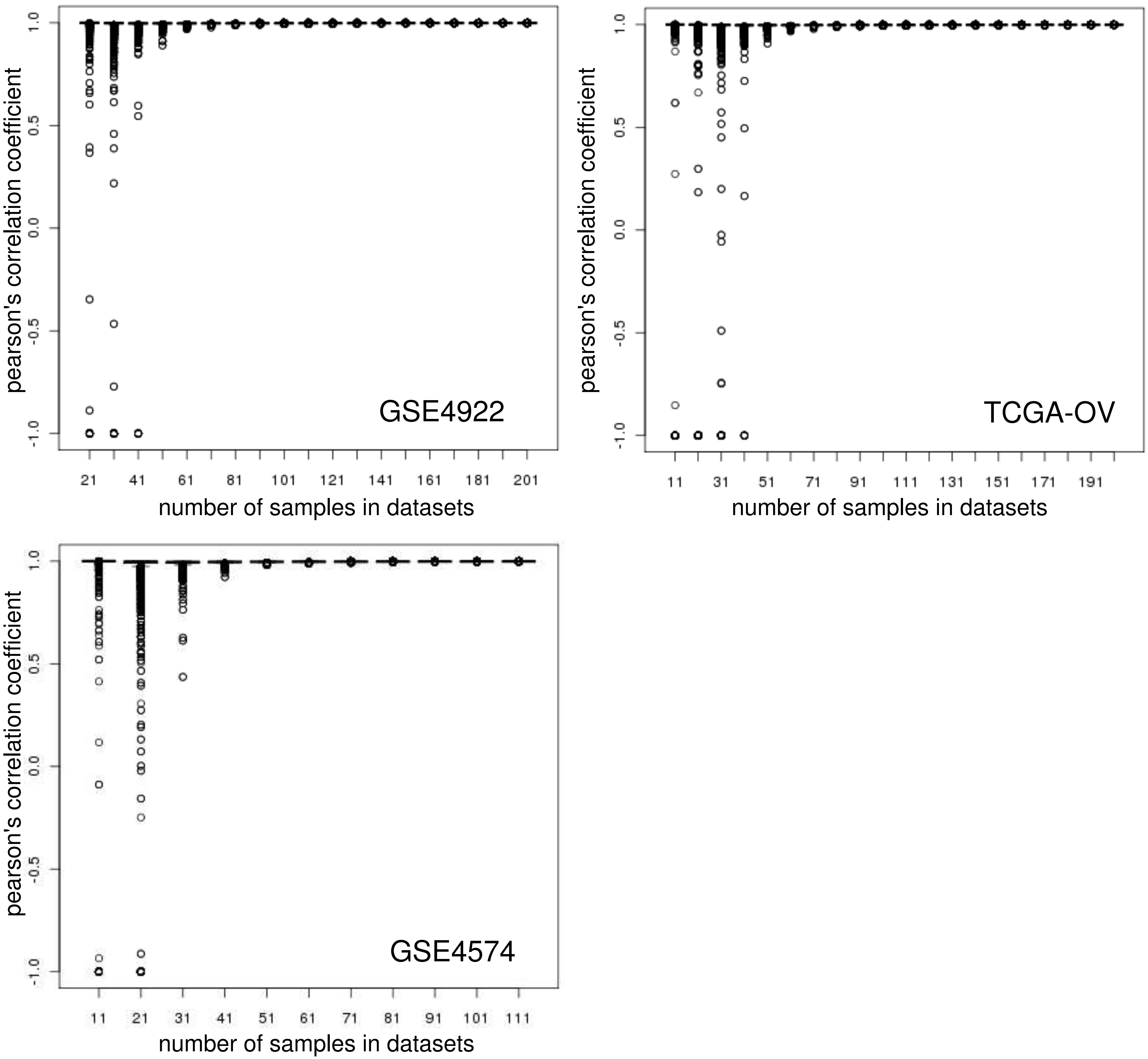

Supplementary figure 5

a)

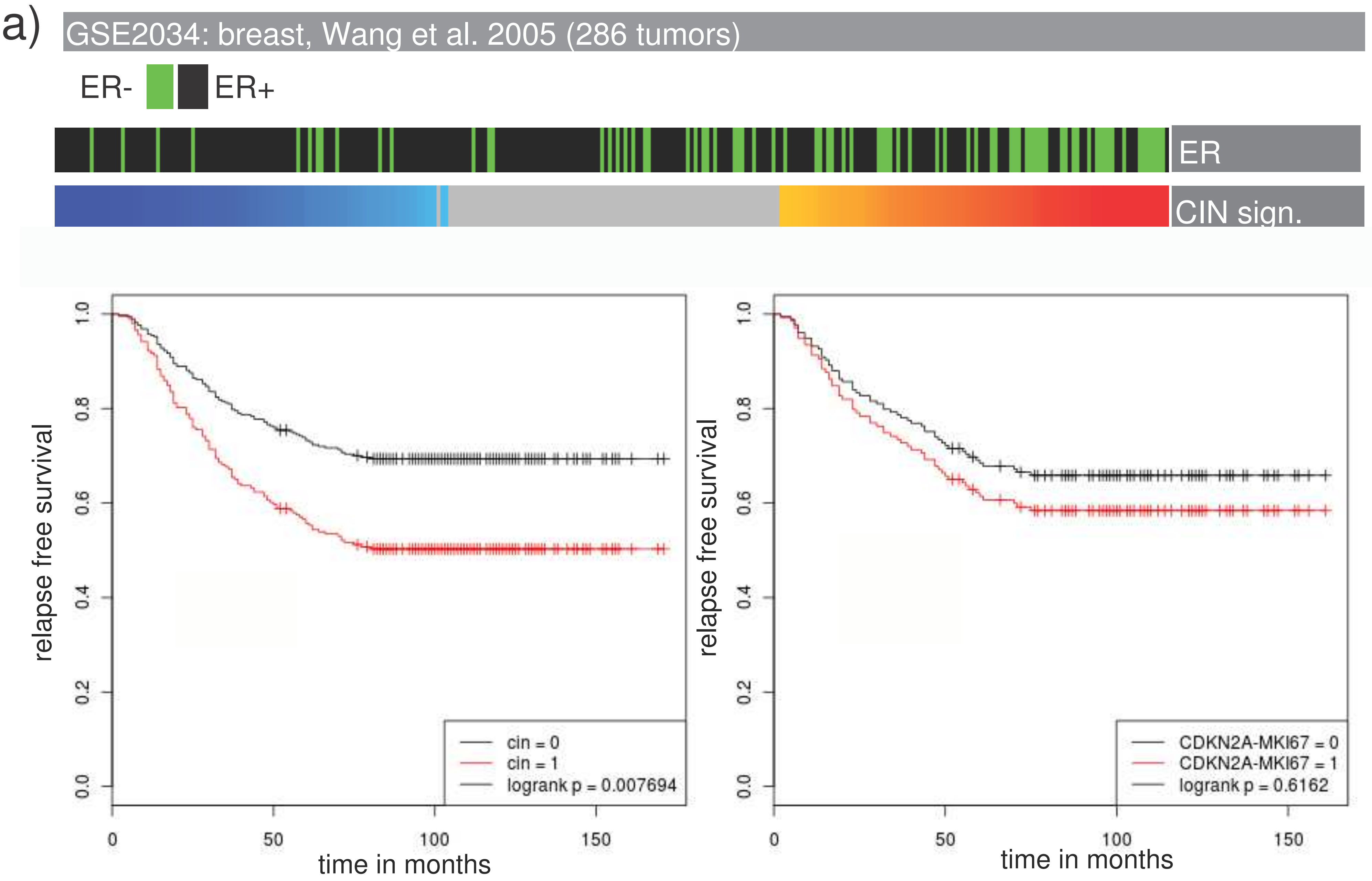

b)

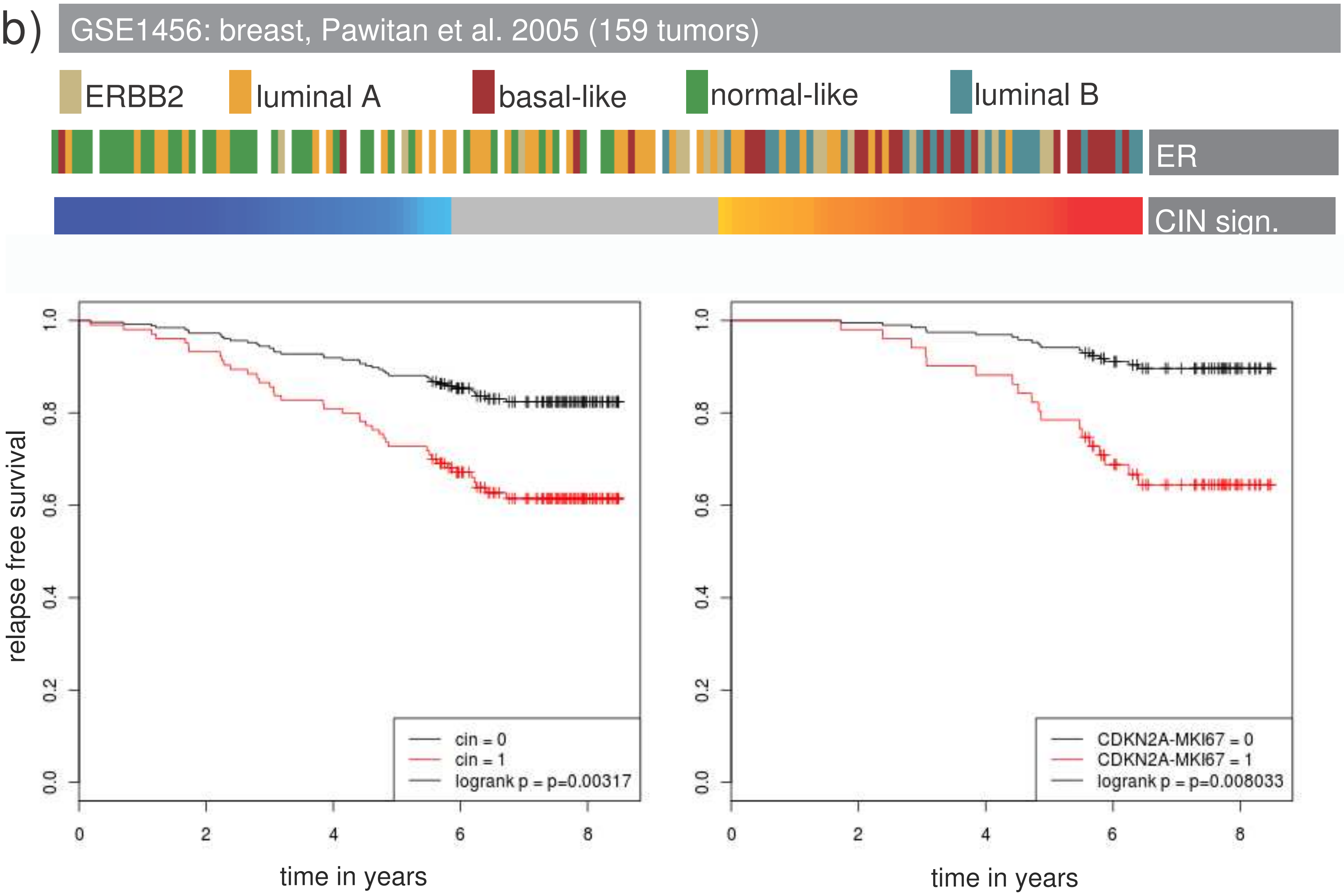

Supplementary Figure 6

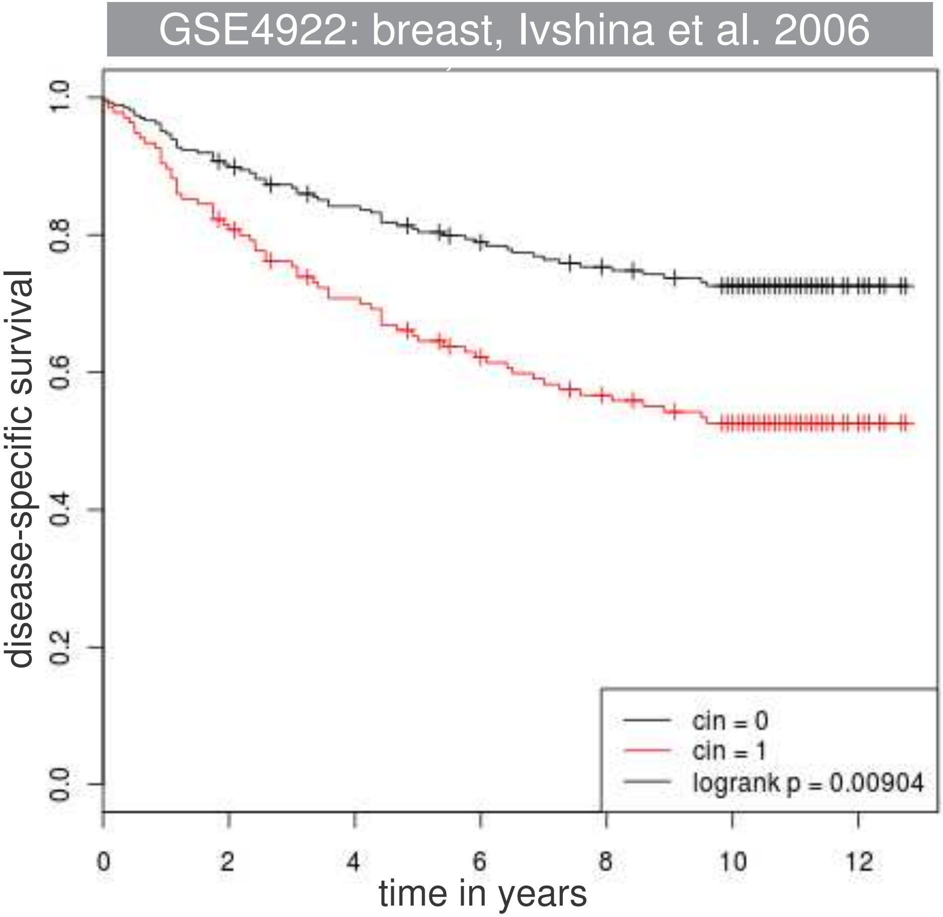

Supplementary figure 7

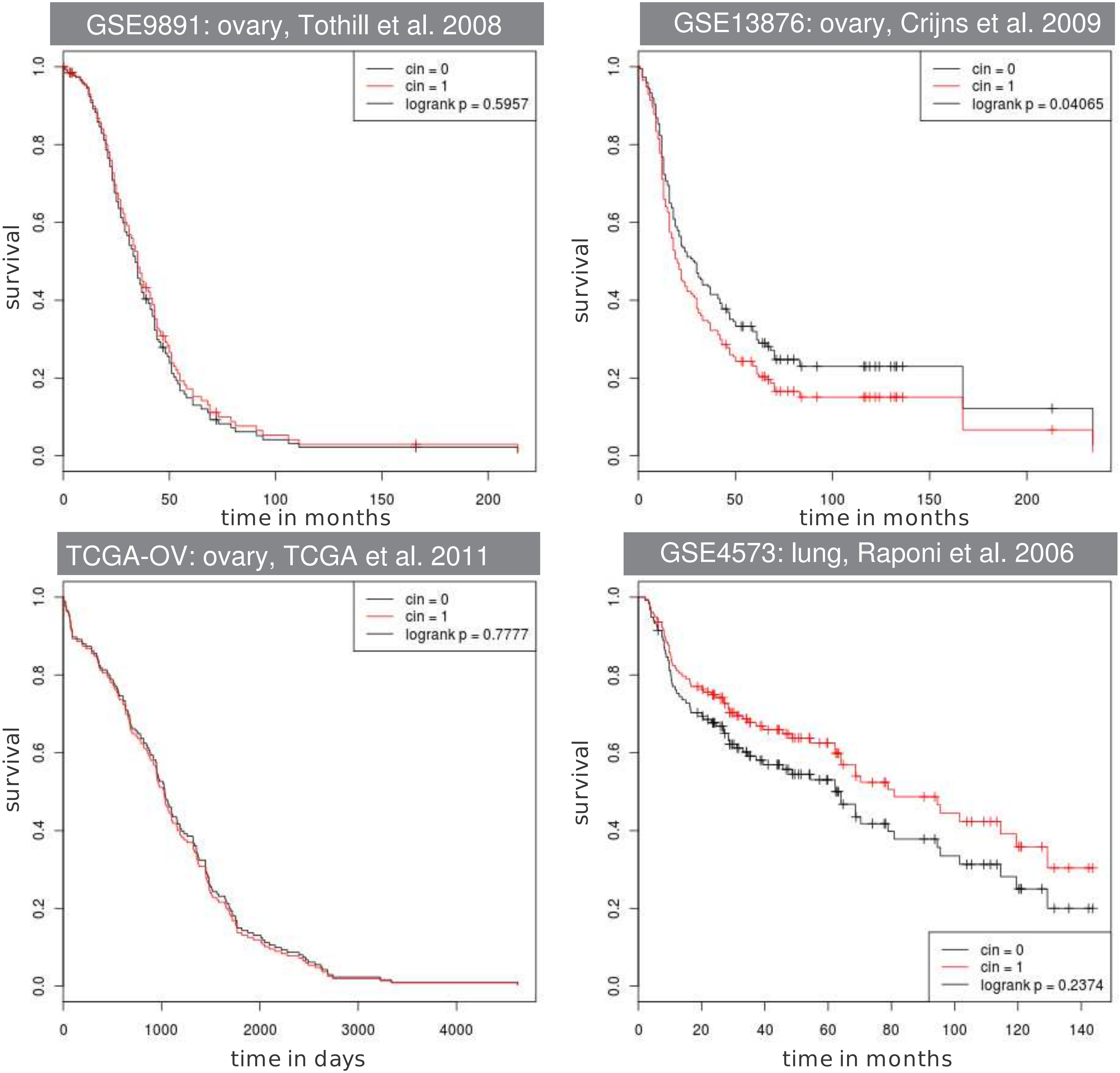

Supplementary figure 7 cont'd

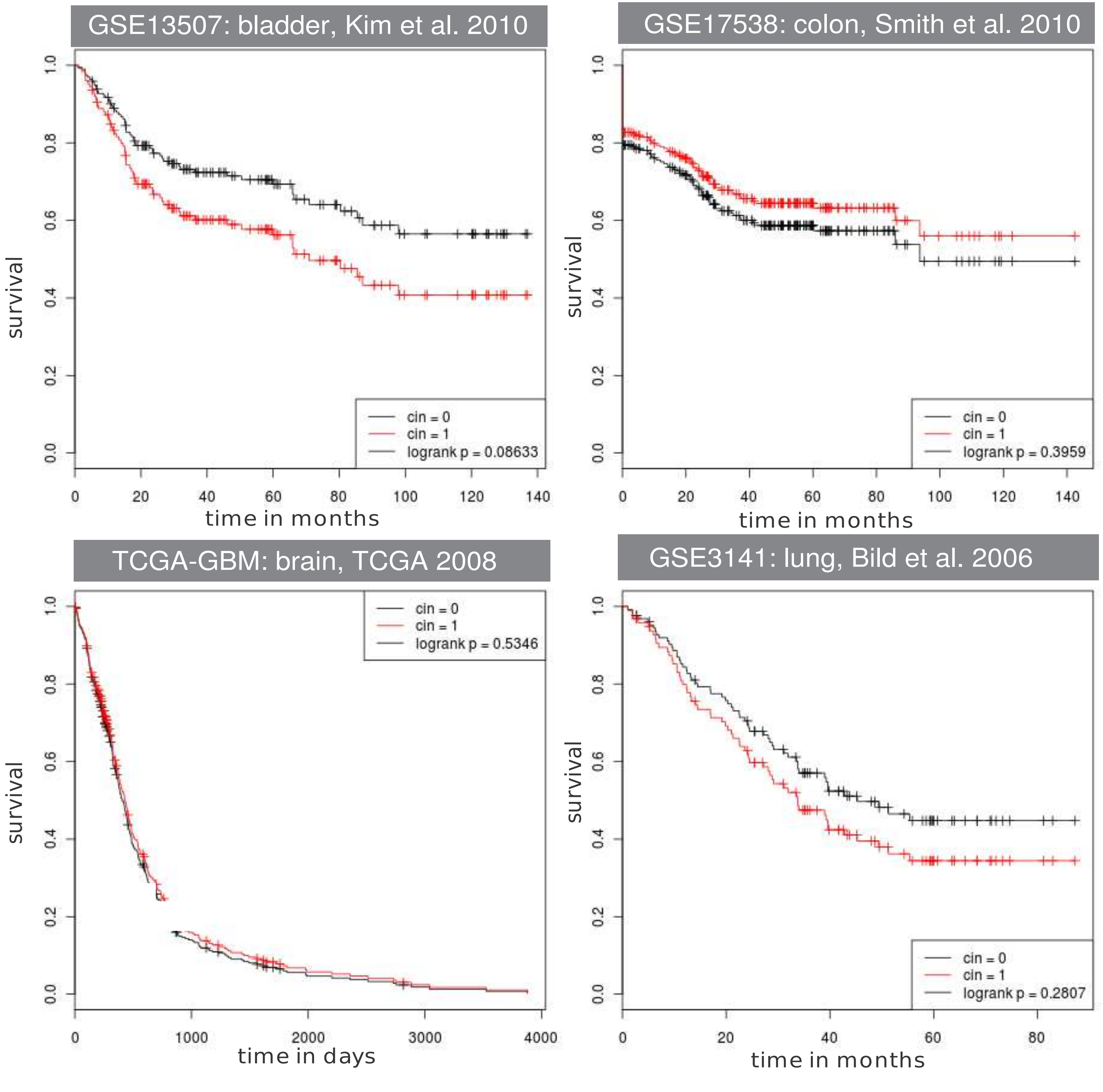

Supplementary Figure 8

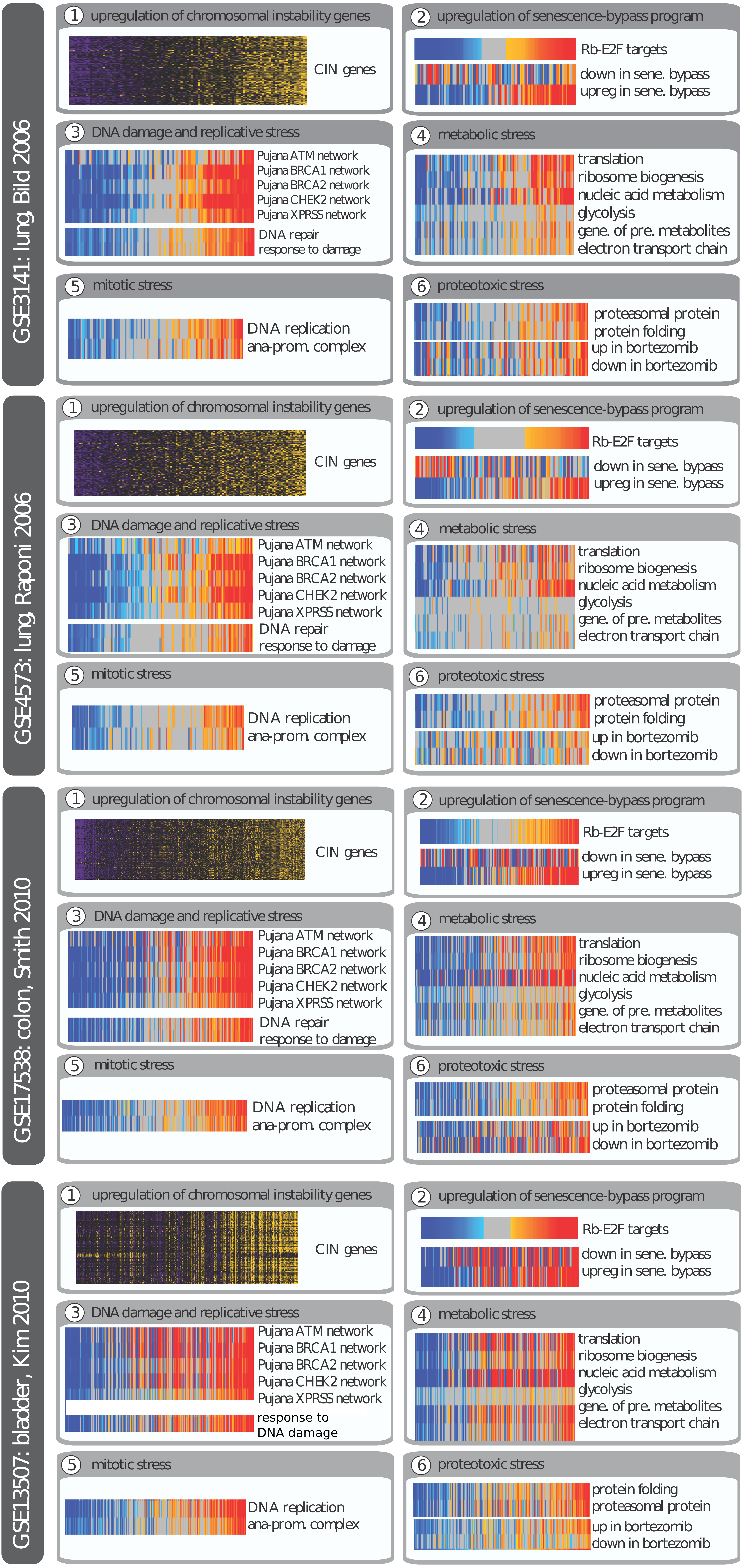

Supplementary Figure 8 cont'd

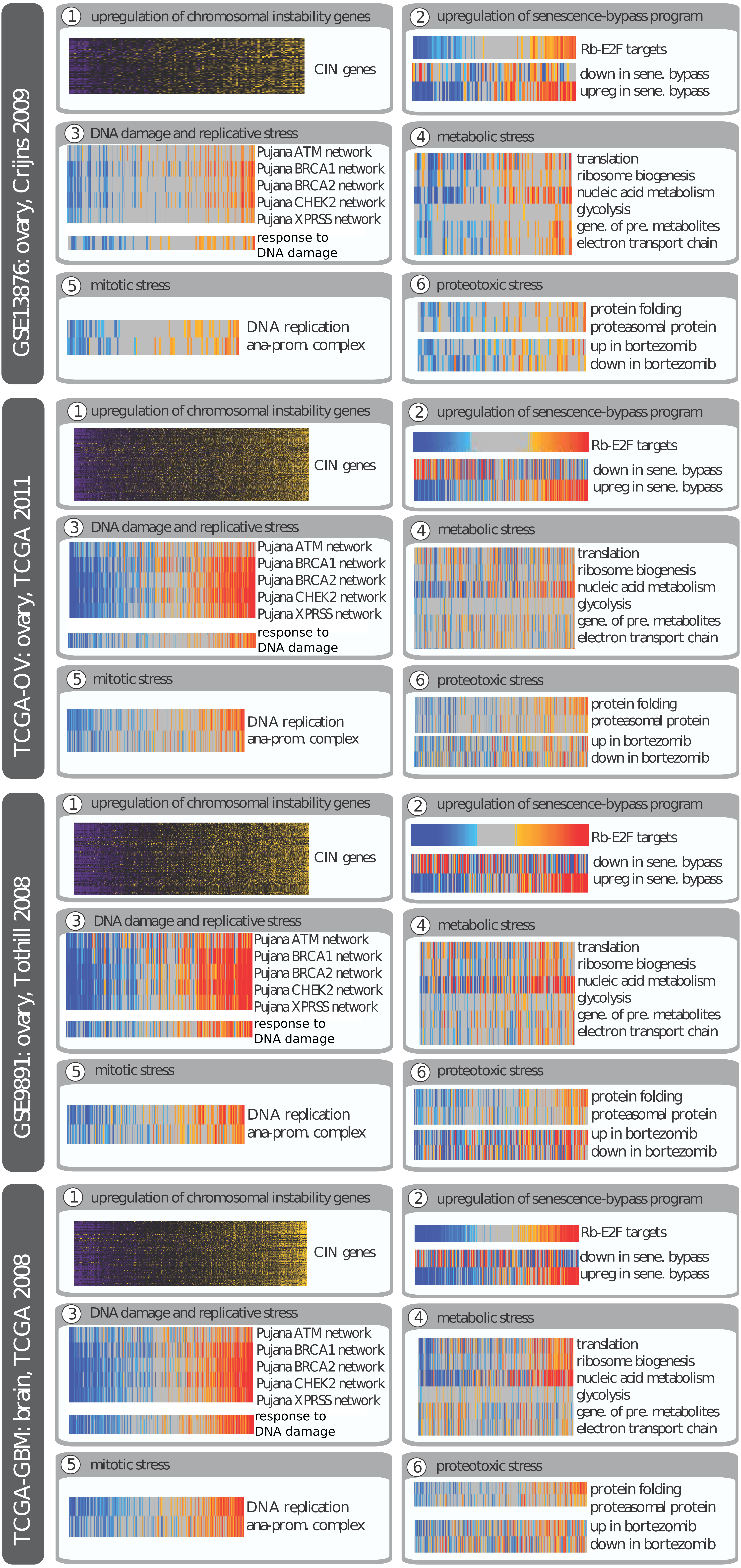

Supplement: Additional file 1 — Supplementary figures. Supplementary figure 1: result of overlap analysis of the modules used. Heat map of the Jaccard indices for overlap analysis among modules used in this study. Pink cells indicate high overlap (Jaccard index ≥ 0.6) while light blue shows no overlap. Supplementary figure 2: comparison of z-score mean and z-score median. Scatter plot of z-scores obtained using mean and median as the test statistic in EA of the Ivshina et al. [21] dataset with the chromosomal instability (CIN) signature. Since the correlation is high and the median is more robust to outliers, it is used for the test statistic. Supplementary figure 3: robustness analysis of SLEA. Step 1: randomization procedure to test for the size of the dataset. Populations of random datasets were created from the three datasets GSE4922 ([GEO:GSE4922]; Ivshina et al. [21]), TCGA-OV (TCGA Nature 2011 [23]) and GSE4573 ([GEO:GSE4573]; Raponi et al. [18]). Each population contained 100 datasets of a fixed number of samples. For GSE4922 [GEO:GSE4922] and TCGA-OV, the sample number varied from 21 to 201, and for GSE4573 [GEO:GSE4573], from 11 to 111. Step 2: for each random dataset in each population, we performed EA with the CIN signature. Step 3: within each population, we performed pair-wise correlation analysis between all random datasets. Step 4: we plotted the distribution of Pearson's correlation values for all populations in a box-and-whisker plot. Correlation values get closer to 1 as sample size increases and are greater than 0.99 for populations of 71 or more. Supplementary figure 4: results of the robustness analyses. Robustness analysis for changes in the cohort was performed for three datasets. Shown here are the plots for them. For GSE4922 [GEO:GSE4922] and TGCA-OV, correlation coefficients for all datasets get closer to 1 as sample size increases. Among all three datasets, correlation is greater than 0.99 for datasets of size 71. Supplementary figure 5: predictive power of the CIN sign [file gm327-S1.PDF]
